# E-*p*-Methoxycinnamoyl-α-l-rhamnopyranosyl Ester, a Phenylpropanoid Isolated from *Scrophularia buergeriana*, Increases Nuclear Factor Erythroid-Derived 2-Related Factor 2 Stability by Inhibiting Ubiquitination in Human Keratinocytes

**DOI:** 10.3390/molecules23040768

**Published:** 2018-03-27

**Authors:** Jiwon Jeong, Lilik Duwi Wahyudi, Young-Sam Keum, Heejung Yang, Jung-Hwan Kim

**Affiliations:** 1 Department of Pharmacology, College of Medicine, Institute of Health Sciences, Gyeongsang National University, Jinju 52727, Korea; jjw8158@naver.com (J.J.); lilik.chemistry_uj@yahoo.com (L.D.W.); 2 Department of Convergence Medical Science (BK21 Plus), Gyeongsang National University, Jinju 52727, Korea; 3 College of Pharmacy, Dongguk University, Goyang 10326, Korea; keum03@dongguk.edu; 4 College of Pharmacy, Kangwon National University, Chuncheon 24341, Korea; heejyang@kangwon.ac.kr

**Keywords:** Nrf2, phenylpropanoid, antioxidant, ubiquination

## Abstract

The nuclear factor erythroid-derived 2-related factor 2 (Nrf2) is a key regulator of gene expression during oxidative stress and drug detoxification. Thus, identifying Nrf2 activators to protect from possible cell damage is necessary. In this study, we investigated whether E-*p*-methoxycinnamoyl-α-l-rhamnopyranosyl ester (MCR), a phenylpropanoid isolated from *Scrophularia buergeriana*, can activate Nrf2 signaling in human keratinocytes (HaCaT). First, we determined the dose- and time-dependent effects of MCR on the expression and activity of Nrf2. The antioxidant response element-luciferase reporter assay and western blot analysis results showed that MCR markedly induced Nrf2 activity and its protein expression, respectively. Further, MCR increased both the mRNA and protein levels of heme-oxygenase-1, one of the Nrf2 target genes, in the cells. Interestingly, we found that Nrf2 stability was remarkably enhanced by MCR. Furthermore, ubiquitin-dependent proteasomal degradation of Nrf2 was significantly reduced by MCR. Thus, MCR might afford skin protection by enhancing Nrf2 stability or by blocking its proteasomal degradation.

## 1. Introduction

Nuclear factor erythroid 2-related factor 2 (Nrf2) is a transcription factor associated with the regulation of oxidative stress and drug detoxification-related genes that are implicated in stress-induced inflammation, cardiovascular disease, aging, and cancer. Under normal conditions, the association of Nrf2 protein with Kelch-like ECH associated protein 1 (Keap1), an inhibitory factor of Nrf2 in the cytoplasm, causes the proteasomal degradation of Nrf2 [1,2]. However, under oxidative or electrophilic stress condition, the proteasomal degradation of Nrf2 is inhibited by dissociation from Keap1 and is released for translocation from the cytoplasm to the nucleus, where it forms heterodimers with a small Maf protein and then binds to the antioxidant response element (ARE) to regulate its target genes [3].

Nrf2 is stabilized through cysteine thiol modification of Keap1 or phosphorylation of Nrf2 at specific serine/threonine residues by several kinases [1] such as mitogen activated protein kinase (MAPK), phosphatidylinositol 3-kinase (PI3K)/Akt, and cyclic-AMP-activated protein kinase α (AMPKα). Many signaling pathways for the regulation of Nrf2 have already been reported [4,5]. MAPK pathways are important for transducing various signals from the cell surface to the nucleus [6]. Many studies have shown that growth factors activated by extracellular signal-regulated protein kinases are associated with the regulation of AREs [7]. Akt has been shown to play a major role in Nrf2 activation and is also known to be involved in the regulation of AREs [8].

AREs are *cis*-acting enhancer sequences found in the promoters of numerous genes such as antioxidant and phase II detoxification enzymes; they are activated in response to oxidants, electrophiles, and natural products [1,9,10,11]. The majority of antioxidant enzymes include heme-oxygenase-1 (HO-1), NAD(P)H:quinine oxidoreductases-1 (NQO-1), and glutamate-cysteine ligase catalytic (GCLC). HO-1 is a major antioxidant enzyme and plays a key role in protecting cells from oxidative stresses and inflammation. These genes are generally regulated by Nrf2; therefore, the Nrf2-ARE pathway is important in the antioxidant system [12,13].

The main function of antioxidant enzymes is to protect cells from damage caused by reactive oxygen species (ROS) such as peroxides, superoxides, and hydroxyl radicals. Numerous studies have shown that ROS cause detrimental effects such as DNA damage, cancer, aging, cell death, and other diseases [14,15]. However, antioxidant compounds might ameliorate these symptoms by scavenging ROS or by regulating antioxidative genes.

The human skin is constantly exposed to ultraviolet (UV) irradiation and ROS, resulting in skin aging or cancer. Therefore, detecting protective compounds against UV and ROS is important [16]. Phenylpropanoids are simple phenolic compounds produced from the aromatic amino acids phenylalanine and tyrosine in plants and are the essential components of structural polymers [17]. They play essential roles in protecting cells from external stresses such as UV irradiation and infections [18]. E-*p*-methoxycinnamoyl-α-l-rhamnopyranosyl ester (MCR) is a phenylpropanoid glycoside isolated from *Scrophularia buergeriana* [16]. Therefore, in the present study, we investigated the mechanism of action of MCR on Nrf2 activation in human keratinocyte cells.

## 2. Results and Discussion

### 2.1. MCR-Induced Growth Inhibition of HaCaT Cells

**Figure 1:**
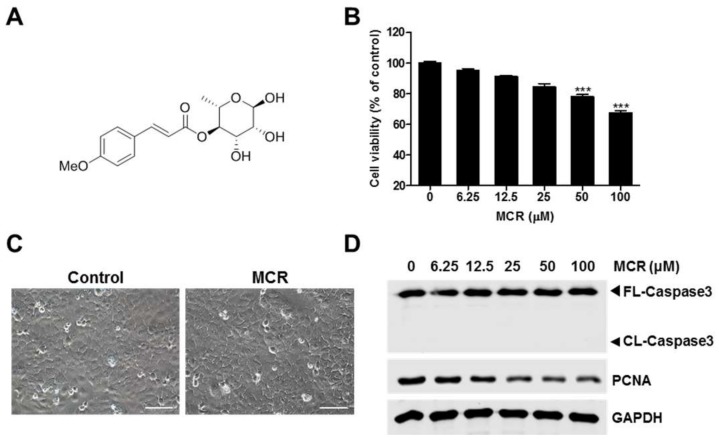
MCR induces minor cell growth inhibition in HaCaT cells. (**A**) Chemical structure of MCR; (**B**) HaCaT cells were treated with indicated concentration of MCR for 24 h, and MTT assay was performed; (**C**) Cells were treated with 50 μM of MCR for 24 h, and live cell images were obtained using a phase contrast microscope; (**D**) Cells were treated with different concentrations of MCR for 24 h, and lysates were subjected to western blot analysis. ***, *p* < 0.0001; Bar, 100 μm.

Because of the lack of cytotoxicity information of MCR (Figure 1A) in HaCaT cells, the cells were treated with different concentrations for 24 h, and MTT assay was performed. The results showed that the cytotoxic effect of MCR increased significantly in a dose-dependent manner (Figure 1B). Whether MCR induced cell death in HaCaT cells was visualized by conducting microcopy observations after MCR (50 μM) treatment for 24 h (Figure 1C). Further, whether MCR induced cell death via the induction of the apoptotic pathway or by inhibiting the cell proliferation pathway was determined. First, cleavage of caspase-3, a marker for apoptosis [19], was measured using western blotting. However, caspase-3 cleavage was not induced by MCR (Figure 1D). Next, the level of proliferating cell nuclear antigen (PCNA), a proliferation marker [20], was measured after treatment with MCR. The level of PCNA decreased in a dose-dependent manner (Figure 1D), suggesting that MCR might affect HaCaT cell proliferation.

### 2.2. MCR Activates Nrf2 Signaling via the ARE System

**Figure 2:**
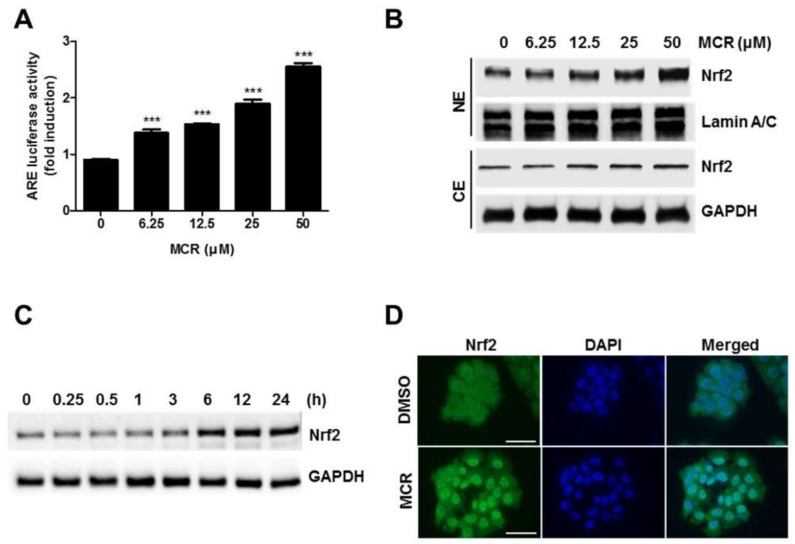
MCR stimulates Nrf2 activation. (**A**) HaCaT-ARE cells were treated with different concentrations of MCR for 24 h and ARE-luciferase activity was measured using a luminometer; (**B**) Cells were treated with different concentrations of MCR for 24 h, and nuclear and cytosolic Nrf2 levels were measured using western blot analysis; (**C**) Cells were treated with 50 μM of MCR for indicated time periods, and whole cell lysates were subjected to western blotting; (**D**) HaCaT cells were treated with 50 μM of MCR for 12 h, and the nuclear localization of Nrf2 (green) was detected using immunocytochemical analysis, as described in the Materials and methods section. Lamin A/C and GAPDH were used as nuclear and cytosolic loading controls, respectively. NE, nuclear extract; CE, cytosolic extract. ***, *p* < 0.0001; Bar, 50 µm.

Because phenylpropanoids are known to be implicated in skin protection, we investigated whether MCR could regulate Nrf2 signaling. Interestingly, we found that MCR remarkably induced the ARE-luciferase activity in the HaCaT-ARE cells (Figure 2A). Next, we measured the level of nuclear Nrf2 protein after treatment with MCR and found that it increased significantly in a dose- and time-dependent manner (Figure 2B,C). Moreover, Nrf2 was significantly accumulated in the nucleus of MCR-treated cells, as revealed by immunocytochemistry analysis (Figure 2D). Because MCR increases Nrf2 accumulation in the nucleus and might induce its target gene expression, investigating the underlying molecular mechanisms is necessary. Two possible mechanisms by which MCR activated Nrf2 could be suggested: first, MCR could activate the signaling pathways by stimulating Nrf2 transcriptional regulation; second, MCR could inhibit the proteasomal degradation of Nrf2 protein. These possible mechanisms were studied in the following experiments.

### 2.3. MCR Induces Nrf2 Target Genes in HaCaT Cells

**Figure 3:**
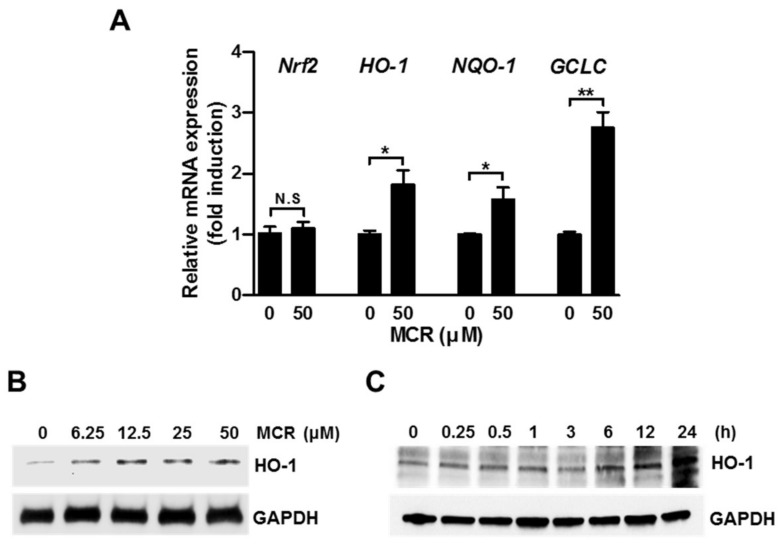
MCR induces Nrf2 target genes in HaCaT cells. (**A**) HaCaT cells were treated with 50 μM of MCR for 12 h, and the expression of Nrf2 target genes was measured using the qPCR method; (**B**) Cells were treated with different concentrations of MCR for 24 h, and lysates were subjected to western blot analysis; (**C**) Cells were treated with 50 μM of MCR for the indicated time periods, and whole cell lysates were subjected to western blotting. GAPDH was used as the loading control. * *p* < 0.05, ** *p* < 0.001; N.S., not significant.

To determine the effects of MCR on the transcriptional regulation of Nrf2, we measured the mRNA level of its target genes such as *HO-1*, *NQO-1*, and *GCLC* by using real-time PCR analysis. MCR significantly increased the mRNA level of each Nrf2 target gene in HaCaT cells (Figure 3A). Moreover, the HO-1 protein level was significantly increased by MCR in a dose- and time-dependent manner (Figure 3B,C). Further, we measured the level of Nrf2 mRNA to determine whether MCR stimulated Nrf2 transcription. However, Nrf2 mRNA level was not affected by MCR, suggesting that MCR might activate Nrf2 by preventing its proteasomal degradation.

### 2.4. MCR Increases the Stability of Nrf2 Protein

**Figure 4:**
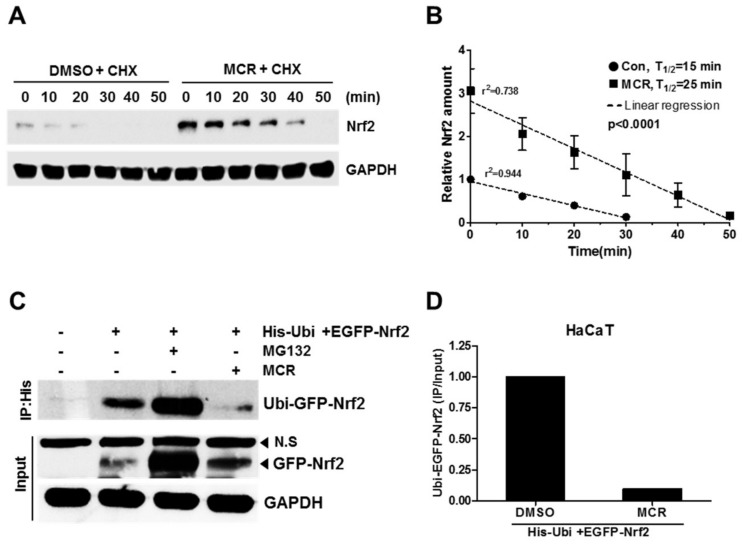
MCR increases Nrf2 stability. (**A**) HaCaT cells were treated with 50 μM of MCR for 10 h before incubation with 5 μg/mL CHX for the indicated times, and whole protein extracts were subjected to western blotting; Nrf2 stability was analyzed using densitometer as shown in (**B**); (**C**) Cells were treated with MCR (50 μM) and/or MG132 (10 μM) for 12 h after cotransfection with pEGFP-Nrf2 and pcDNA3.1-His ubiquitin. His-ubiquitinated proteins were purified using Ni-NTA agarose beads, and uibiquitinylated GFP-Nrf2 was analyzed using western blotting; The amount of uibiquitinylated GFP-Nrf2 was measured using a densitometer as shown in (**D**). CHX, cycloheximide; Ubi, ubiquitin.

Several Nrf2 inducers or activators are known to suppress ubiquitin-dependent degradation of Nrf2 by preventing its proteasomal degradation [1,13,21]. To confirm whether MCR affected Nrf2 protein stability, we treated the cells with cycloheximide after incubation with MCR. Nrf2 accumulation mediated by MCR lasted up to 40 min (Figure 4A). Further, the half-life of Nrf2 protein was assessed (Figure 4B). The Nrf2 half-life was significantly prolonged by MCR from 15 min to 25 min. Furthermore, to investigate whether MCR suppressed Nrf2 ubiquitination, we used the Ni-NTA purification system to purify His-ubiquitinylated-Nrf2. The GFP-Nrf2 ubiquitination was remarkably decreased when the HaCaT cells were treated with MCR (Figure 4C,D). These data indicate that MCR suppressed GFP-Nrf2 ubiquitination, and vice versa, and increased Nrf2 protein stability by inhibiting its ubiquitination. Several studies have shown that MAPK, PI3K/Akt, and AMPKα are involved in the translocation of Nrf2 to the nucleus [4]. To analyze whether these pathways were involved, we used specific inhibitors for MAPK, PI3K/Akt, and AMPKα. However, their pharmacological inhibitors failed to affect MCR-induced Nrf2 translocation to the nucleus. Our results showed that MCR did not change the accumulation of Nrf2 via the phosphorylation of MAPK, PI3K/Akt, and AMPKα (Figure S1). Nrf2 is an unstable protein, and its half-life is very short and regulated by the 26S proteasome [13,22]. Keap1 acts as a substrate for the ubiquitination of Nrf2 in Cul3-dependent E3 ubiquitination systems. However, Nrf2 activators/inducers can increase Nrf2 stability. Mangiferin, a compound extracted from the mango plant, was also found to increase Nrf2 protein stability and inhibit Nrf2 ubiquitination [21].

In conclusion, our study confirms that MCR increases Nrf2 accumulation in the nucleus and its subsequent binding to ARE and upregulates the expression of HO-1. This occurs via the inhibition of its ubiquitination and degradation in HaCaT cells. Because MCR stabilizes Nrf2 without changing the upstream Nrf2 signaling, it can be applicable for chemoprevention or skin protection.

## 3. Materials and Methods

### 3.1. Cell Culture and Reagents

The human keratinocyte cells (HaCaT) were obtained from the American Type Culture Collection (ATCC, Manassas, VA, USA). HaCaT and HaCaT-ARE (stably transfected with 3xARE-luciferase gene) [23] cells were maintained at 37 °C in a humidified atmosphere of 5% CO_2_/95% air in RPMI1640 supplemented with 10% heat-inactivated FBS and 50 U/mL penicillin/streptomycin mixture (Invitrogen, Carlsbad, CA, USA). MCR was kindly provided by Dr. Heejung Yang of the Kangwon National University. For western blotting, anti-Nrf2 (Cat: ab137550, Abcam, Cambridge, MA, USA), anti-HO-1 (Cat: ab68477, Abcam), anti-Lamin A/C (Cat: sc20681, Santa Cruz Biotechnology, Santa Cruz, CA, USA), and GAPDH (Cat: sc25778, Santa Cruz Biotechnology) were used.

### 3.2. Cell Viability Assay

The effect of MCR on the viability of HaCaT cells was determined using the MTT assay [24]. Briefly, the cells were treated with various concentrations of MCR for 24 h. After incubation, 20 μL of MTT stock solution (5 mg/mL) was added to each well. After incubation for 2 h, the formazan crystals in each well were dissolved in 200 μL DMSO and measured at 570 nm by using an ELISA plate reader (Tecan infinite 200, Salzburg, Austria).

### 3.3. Luciferase Reporter Gene Assay

HaCaT-ARE cells were treated with various concentrations of MCR or DMSO alone as a control for 24 h. After 24 h incubation, cells were extracted with 100 μL of passive lysis buffer (Promega, San Luis Obispo, CA, USA) for 20 min at room temperature. The ARE-luciferase activity was measured in a luminometer by using the Dual-Luciferase Reporter Assay System according to manufacturer’s instructions, after the total protein concentration was normalized.

### 3.4. Western Blot Analysis

Cells were treated for different times with the indicated compounds or vehicle as a control, and then extracted using RIPA buffer [25 mM Tris-HCl (pH7.6), 150 mM NaCl, 1% NP40, 1% sodium deoxycholate, 0.1% SDS] containing a protease inhibitor cocktail on ice. Cells were sonicated for 10 s at 20% of the full power, followed by centrifugation at 14,500 *g* for 15 min at 4 °C. The nuclear and cytosolic protein extracts were prepared according to the modified method as described previously (citation). Nuclear proteins and cytoplasmic fractions were extracted using M-PER mammalian lysis buffer (Thermo Fisher Scientific, Waltham, MA, USA) containing protease inhibitors. The supernatants were used as cytoplasmic fractions. The nuclear pellets were resuspended in M-PER buffer and sonicated for 10 s at 20% of the full power and then centrifuged at 14,500 *g* for 10 min at 4 °C. The protein concentrations were measured using bicinchoninic acid reagent. Proteins were separated electrophoretically on 7.5–10% SDS-polyacrylamide gels. Blots were transferred onto nitrocellulose membranes (Bio-Rad, Hercules, CA, USA) and then blocked with 5% non-fat dry milk in PBS or TBS containing 0.1% Tween 20 for 1 h and incubated with primary antibodies overnight at 4 °C. After incubation with the secondary antibody at room temperature for 1 h, chemiluminescence was detected using the ECL substrate solution.

### 3.5. Immunocytochemistry

The nuclear translocation of Nrf2 was assessed by incubating the cells with or without MCR for 12 h in cover-slip plates. The cells were fixed with 4% paraformaldehyde for 10 min and cold methanol for 2 min and washed three times with PBS containing 0.3% Triton X-100 (PBST). Cells were incubated with 5% donkey serum (Abcam) in PBST, washed with 0.3% PBST, and then incubated with anti-Nrf2 antibody (1:200) in 3% BSA-PBST for overnight at 4 °C. The slides were additionally incubated with Alexa Fluor^®^ 488 for 1 h and mounted with DAPI (0.125 μg/mL) in PBS for 1 min. Stained cells were analyzed using fluorescence microscopy by using oil immersion at 40× (ECLIPSE Ti-U, Nicon, Tokyo, Japan).

### 3.6. Real Time-PCR Analysis

Total RNA was extracted from the cells by using TRIzol reagent (Invitrogen) according to manufacturer’s instruction. Next, cDNA was synthesized from 1 µg of total RNA using SuperScript™ II reverse transcriptase (Invitrogen), according to manufacturer’s instruction. Real-time quantitative PCR was performed to quantify the mRNA level of Nrf2 and its target genes. Reactions were performed using 10 μL SYBER Green PCR Master Mix (Roche, Mannheim, Germany) with 25 ng cDNA at 95 °C for 5 min for polymerase activation, at 95 °C for 10 s for denaturation, at 60 °C for 10 s for annealing, at 72 °C for 20 s for extension, and 10 s at 40 °C for cooling. The following quantitative PCR oligonucleotide primer sets were used: Nrf2 forward, 5′-TCT TGC CTC CAA AGT ATG TCA A-3′ and reverse, 5′-ACA CGG TCC ACA GCT CAT C-3′ and HO-1 forward, 5′-GAG TGT AAG GAC CCA TCG GA-3′ and reverse, 5′-GCC AGC AAC AAA GTG CAA G-3′ and NQO-1 forward, 5′-TCC TTT CTT CTT CAA AGC CG-3′, and reverse, 5′-GGA CTG CAC CAG AGC CAT-3′ and GCLC forward, 5′-CTT TCT CCC CAG ACA GGA CC-3′ and reverse, 5′-CAA GGA CGT TCT CAA GTG GG-3′ and GAPDH forward, 5′-AAG GTG AAG GTC GGA GTC AA-3′ and reverse, 5′-AAT GAA GGG GTC ATT GAT GG-3′. The *Nrf2*, *HO-1*, *NQO-1*, *GCLC*, and *GAPDH* genes were amplified for 45 cycles. All experiments were triplicated.

### 3.7. Protein Stability Assay

First, Nrf2 protein degradation was tested by treatment with cycloheximide (CHX). HaCaT cells were treated with or without 50 μM MCR for 10 h and incubated with CHX (5 μg/mL) for different time intervals. Further, the protein amount of Nrf2 was measured using western blot, and the half-life of Nrf2 was measured using densitometry.

For different experiments, HaCaT cells were transfected with pcDNA4-His-Ubi (4 μg) and pEGFP-Nrf2 (4 μg) for 25 h by using linear polyethylenimine (PEI) reagent and treated with MG132 (10 μM; Merck, Billerica, MA, USA) and/or MCR (50 μM) for 12 h. Next, the cells were harvested using RIPA lysis buffer for whole protein. Whole cell extract (500 μg) was incubated with 100 μL of Ni-NTA beads for 1 h at 4 °C in a rotary shaker. After washing 3 times with the same buffer for 5 min at 4 °C in a rotary shaker, bead–protein complexes were centrifuged at 1000× *g* for 1 min. The beads were resuspended with 65 μL of 2× Laemmli sample buffer and boiled for 5 min, and the samples were subjected to western blotting.

### 3.8. Statistical Analysis

Experimental values were expressed as mean ± SD. Statistical analysis was performed using two-tailed Student’s *t*-test for unpaired data, with *p* < 0.05 considered statistically significant.

## Supplementary Materials

The following are available online, Figure S1: MAPK and AKT have no effect on the Nrf2 signals.
