# Supplementary material for: E-p-Methoxycinnamoyl-α-l-rhamnopyranosyl Ester, a Phenylpropanoid Isolated from Scrophularia buergeriana, Increases Nuclear Factor Erythroid-Derived 2-Related Factor 2 Stability by Inhibiting Ubiquitination in Human Keratinocytes
Source: Molecules. 2018 Mar 27;23(4):768. doi: 10.3390/molecules23040768 (PMC6017950; doi:10.3390/molecules23040768)
Supplement: Supplementary file 1 [file molecules-23-00768-s001.pdf]

Supplementary Figure 1

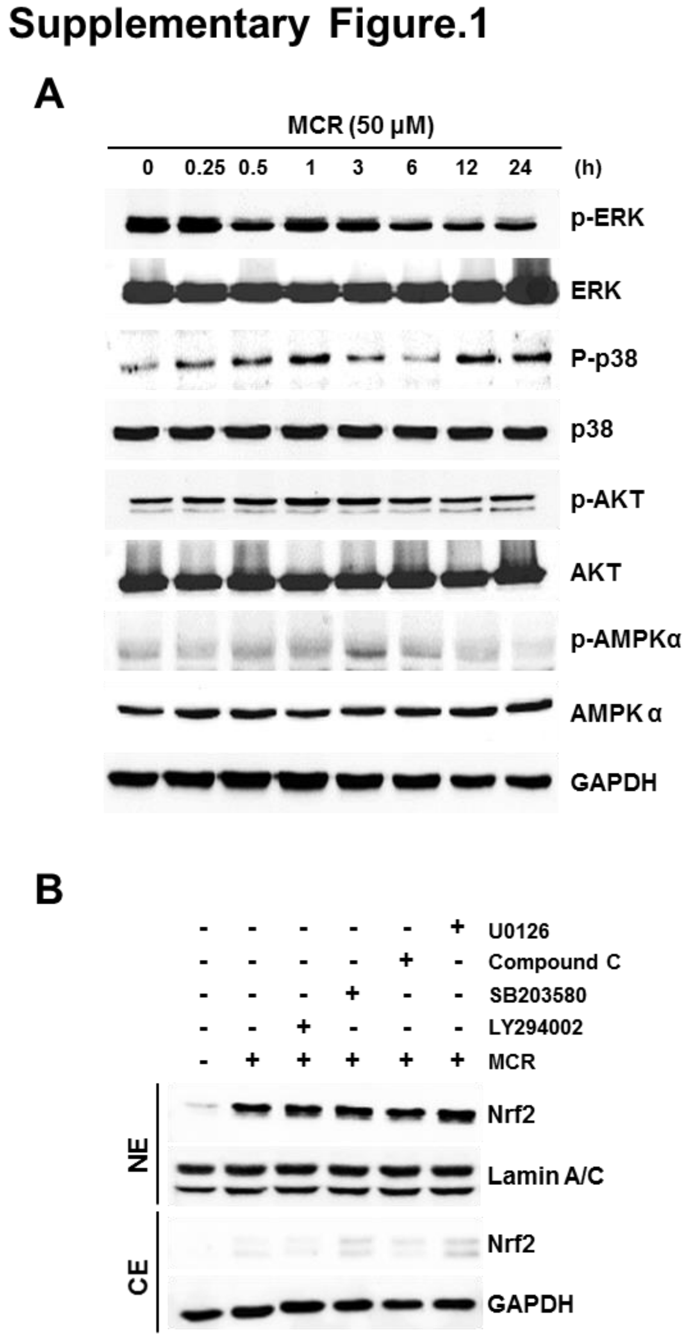

Sppl. Fig 1. MAPK and AKT have no effect on the Nrf2 signals (A) HaCaT cells were treated with 50 μM of MCR for indicated times and whole cell lysates were subjected to Western blotting. (B) Cells were treated with 20 μM of each U0126 (Erk inhibitor), SB203580 (p38 inhibitor), LY294002 (PI3K inhibitor) or Compound C (AMPK inhibitor) for 12 h in the presence of 50 μM of MCR and then nuclear and cytosolic levels of Nrf2 were measured by Western blot analysis. NE, nuclear extract; CE, cytosolic extract.
